# *WingMesh*: A Matlab-Based Application for Finite Element Modeling of Insect Wings

**DOI:** 10.3390/insects11080546

**Published:** 2020-08-18

**Authors:** Shahab Eshghi, Vahid Nooraeefar, Abolfazl Darvizeh, Stanislav N. Gorb, Hamed Rajabi

**Affiliations:** 1 Functional Morphology and Biomechanics, Institute of Zoology, Kiel University, 24118 Kiel, Germany; sgorb@zoologie.uni-kiel.de (S.N.G.); harajabi@hotmail.com (H.R.); 2 Faculty of Mechanical Engineering, University of Guilan, Rasht 4199613776, Iran; vahid.nooraeefar@gmail.com (V.N.); adarvizeh@guilan.ac.ir (A.D.)

**Keywords:** biological structures, computer vision, mesh generation, simulation, digital image processing

## Abstract

**Simple Summary:**

Manual modeling of complicated insect wings presents considerable practical challenges. To overcome these challenges, therefore, we developed *WingMesh*. This is an application for simple yet precise automatic modeling of insect wings. Using a series of examples, we showed the performance of our application in practice. We expect *WingMesh* to be particularly useful in comparative studies, especially where the modeling of a large number of insect wings is required within a short time.

**Abstract:**

The finite element (FE) method is one of the most widely used numerical techniques for the simulation of the mechanical behavior of engineering and biological objects. Although very efficient, the use of the FE method relies on the development of accurate models of the objects under consideration. The development of detailed FE models of often complex-shaped objects, however, can be a time-consuming and error-prone procedure in practice. Hence, many researchers aim to reach a compromise between the simplicity and accuracy of their developed models. In this study, we adapted *Distmesh2D*, a popular meshing tool, to develop a powerful application for the modeling of geometrically complex objects, such as insect wings. The use of the burning algorithm (BA) in digital image processing (DIP) enabled our method to automatically detect an arbitrary domain and its subdomains in a given image. This algorithm, in combination with the mesh generator *Distmesh2D*, was used to develop detailed FE models of both planar and out-of-plane (i.e., three-dimensionally corrugated) domains containing discontinuities and consisting of numerous subdomains. To easily implement the method, we developed an application using the Matlab App Designer. This application, called *WingMesh*, was particularly designed and applied for rapid numerical modeling of complicated insect wings but is also applicable for modeling purposes in the earth, engineering, mathematical, and physical sciences.

## 1. Introduction

The finite element (FE) method is a numerical technique which is generally used to simulate a physical phenomenon in the virtual world by solving complex boundary value problems [1,2]. FE software packages were developed to simplify often complicated simulation processes. They are especially very common in engineering applications [3,4,5] and are becoming increasingly popular in the investigation of the mechanical behavior of biological structures, such as complex human and animal body parts [6,7,8,9,10,11,12,13].

Although providing a user with a high degree of flexibility, all available FE packages have a common need: an accurate model. A model is a domain which is subdivided into smaller polygonal or polyhedral meshes, so-called elements [14]. A modeling process, however, may present many challenges and can be rather time-consuming, especially when dealing with complex geometries [15,16,17], which is usually the case in biology. The skills of the software user can also strongly influence the process and the final result. These often lead to oversimplified models and, therefore, can affect the accuracy of simulation results. How can this problem be overcome?

In 2004, Persson and Strang aimed to address this problem by developing a simple meshing technique called *Distmesh2D* [18]. As intended by its developers, the method, which was implemented in Matlab code, provided an effective tool to mesh a given domain automatically. The simplicity of the method and the high quality of the produced mesh are the key advantages of the proposed method. However, it also has a major drawback: finding the distance to boundaries by the use of the mathematical equation *f*(*x*, *y*) = 0 or by values of a discrete set of points, as explained by the authors, is a time-consuming and error-prone procedure for complex geometries. Due to the use of a mathematical scheme to define the distance function (see Section 2.5), *Distmesh2D* also has limitations when meshing a domain containing discontinuities.

Here, we aimed to address these challenges and improve the performance of *Distmesh2D* but still maintain its simplicity. To this end, we used computer vision to automatically detect the boundary of a domain in a given image. We combined it with the mesh generator *Distmesh2D* to develop an application for the rapid modeling of geometrically complex domains that consist of several subdomains. The applicability of the method is not limited to in-plane domains, but it can also mesh out-of-plane (i.e., corrugated) objects. We specifically designed and used our method to develop models of insect wings. The proposed application, called *WingMesh*, draws extensively on Persson and Strang’s account in an attempt to offer a simple, but more practical, meshing tool.

## 2. Materials and Methods

The modeling method presented in this study, called *WingMesh*, consists of several algorithms that interact with each other. The method requires an input image to identify the boundaries of a given domain as well as subdomains within that. The code *Distmesh2D* is then employed to mesh the identified domain. Other algorithms were added to the main algorithms to model out-of-plane domains and create a *.inp file, which is the Abaqus input file format [19]. The key parts of the developed Matlab code and the full code of the method, together with a graphical user interface (GUI) (see Section 3), are accessible in the Supplementary Materials (Codes S1 and S3, Method S1).

### 2.1. Burning Algorithm for Detection of the Boundary of a Given Domain

**Figure 1:**
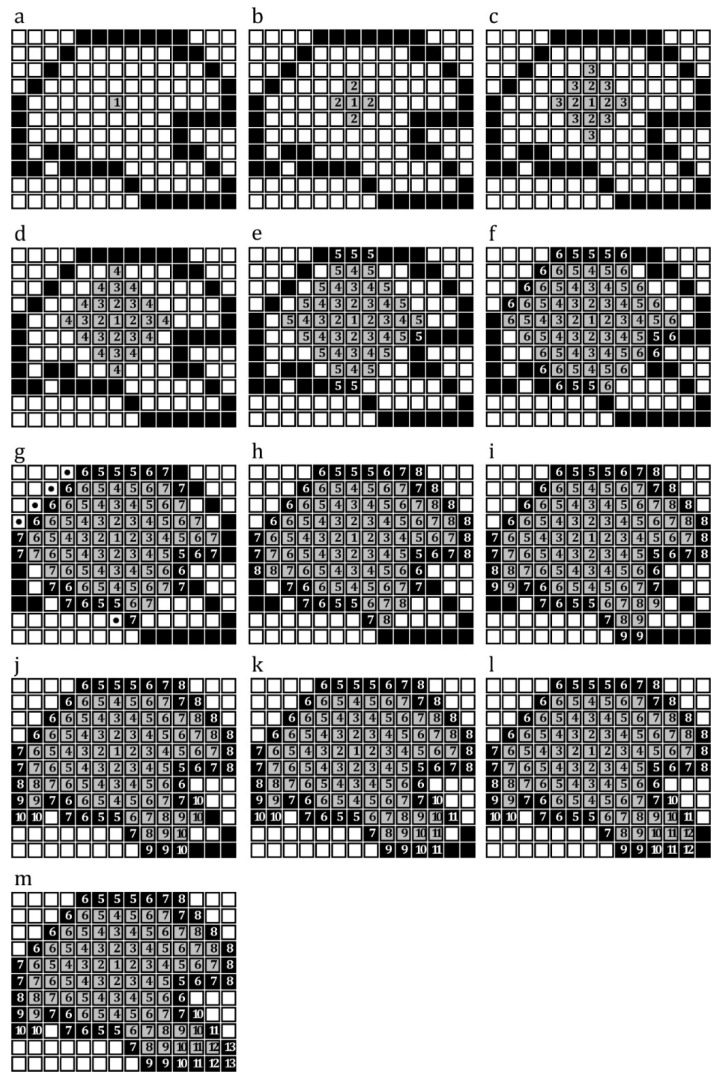
Detection of the border of an arbitrary domain using the Burning Algorithm. (**a**) A white pixel inside the domain is selected. (**b**) The BA searches for white and black pixels around the selected pixel in four orthogonal directions. (**c**) The BA searches for white and black pixels around the detected white pixels in the previous iteration. (**d**–**m**) This process continues until there is no white pixel inside the domain (**m**).

The burning algorithm (BA) was used to extract the boundary of a domain in a given image [20,21]. The BA needs a digital black and white image as the input, in which black pixels, with the pixel value of 0, represent the border of the domain and white pixels, with the pixel value of 1, represent regions that are situated inside and outside of the domain. The BA uses the matrix of the input image to detect the boundary of the domain in that image. This process starts with choosing a pixel within the domain by the user (Pixel 1 in Figure 1a) and continues by detecting white pixels around the selected pixel. To this end, the algorithm checks the colors of pixels located in the four orthogonal directions of the selected white pixels (Pixels 2 in Figure 1b). The coordinates of the found black pixels are stored in a matrix, and the colors of detected white and black pixels are changed to 0.8 (light grey) and 0.1 (dark grey), respectively, in order to avoid their reselection in the next iteration. This process continues by searching for white and black pixels around only white pixels detected in the previous iteration (Figure 1c–m). This process continues until all white pixels inside the domain are detected (Figure 1m). Code S2 and Video S1 in the Supplementary Materials are the source code of BA and a simple illustration of how it works, respectively.

### 2.2. Detection of Subdomains within a Given Domain

The function BA can also detect subdomains within a given domain. When the main domain contains any subdomain, the application first finds the white pixels outside the domain. This process eventually results in the detection of the boundary of the domain.

To find the boundary of each subdomain, the user should select a pixel inside that subdomain in the input image. By this, the BA finds the pixels located on the boundary of the subdomain using the same method explained earlier. This process continues as long as the user selects a pixel in a new subdomain.

### 2.3. Detection of Discontinuities in a Given Domain

The function BA can detect any discontinuity, such as holes, cracks, etc., in a given domain. For this purpose, if any discontinuity exists, the user should select a pixel in each discontinuity in the input image (Video S2). After this, using the same method as described before, BA finds the boundary of each selected discontinuity. By this, the application detects discontinuities and excludes them from the main domain. To this end, after meshing the structure, the application finds all elements inside the region of discontinuity and excludes them from the model.

### 2.4. Development of a Corrugated Model

In a recent study, we developed a method for modeling out-of-plane (i.e., corrugated) domains [22]. Here, we modified this technique to make it more efficient and easier to implement. This technique requires an additional input image with the same frame size as the main input image. The other image should include information regarding the corrugation of the out-of-plane domain. The information should include the location of the maximum and minimum heights, indicated by the black and white colors, respectively. The value of pixels in the secondary image, therefore, serves as a measure of the height of that pixel: pixel values 0 and 1 indicate the maximum and minim heights, respectively. If there is more than one maximum or minimum height in a domain, any local extremum can be marked in grey color. The intensity of the grey color in each local extremum indicates the relative height of that extremum compared to the absolute extremum.

The recursive Equation (1) is used to smooth the corrugations to avoid any abrupt change in the height of a model at the location of an extremum.

(1) $$v\left(r.c\right)=mean\left(\overset{1}{\underset{i=-1}{\sum }}\overset{1}{\underset{j=-1}{\sum }}v\left(r+i.c+j\right)\right)$$

where r and c represent the number of the row and column of a pixel in the image, respectively. v is the value of that pixel. *i* and *j* are the index of the row and column of the pixels in the image. This equation recursively updates the color intensity of the pixels in the secondary image and, thereby, the height of those pixels in the model. The number of iterations, which is set by the user, controls the sharpness of corrugations in the developed model. The values of pixels in the secondary input image, which are between 0 and 1, represent the relative heights of corrugations.

**Figure 2:**
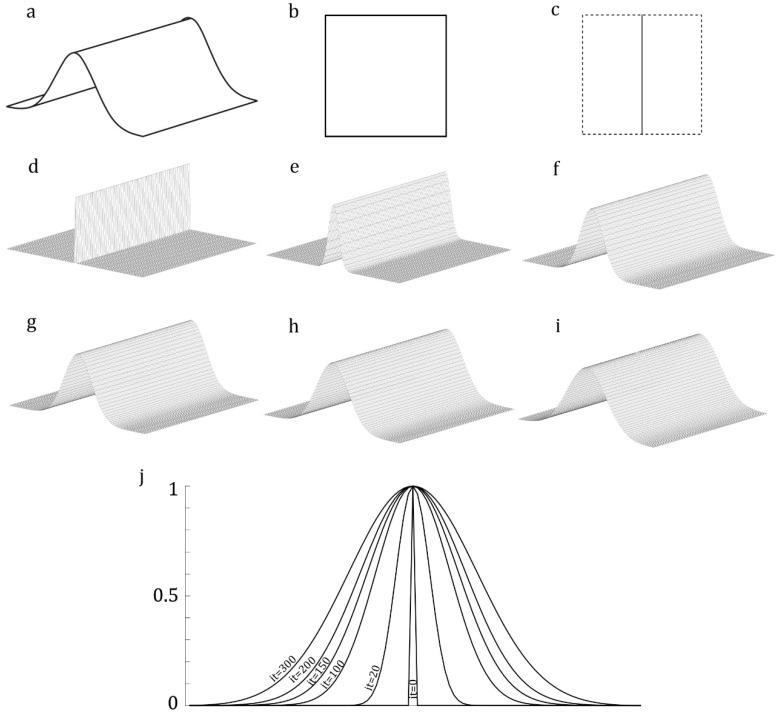
Modeling of an out-of-plane domain. (**a**) An out-of-plane domain. (**b**) A top view image of the domain. The image is used by the BA to detect the domain. (**c**) The secondary image contains a black line that represents the maximum height. The regions with zero height are colored in white. (**d**) A developed model based on the input images. (**e**–**i**) Smoothing the height of the meshed model using the iterative algorithm. (**j**) Changes in the corrugation pattern in different iterations.

Figure 2a shows a corrugated object. The image of the object from the top view is shown in Figure 2b. Figure 2c shows the secondary input image, which has the same frame size as the image shown in Figure 2b. The black line in the middle of the image represents the position of the only available height maximum, and the white color corresponds to the regions with the minimum height. When using these two images, the application develops a model similar to that shown in Figure 2d. Figure 2j shows the gradual changes in the corrugation of the model by the use of Equation (1) after 20, 100, 150, 200, and 300 iterations (Figure 2e–i).

### 2.5. Mesh Generation

*Distmesh2D* is a mesh generator in Matlab which employs a distance function, *d*(*x*,*y*), to describe the geometry of a domain [18]. The Delaunay algorithm is used in *Distmesh2D* to generate triangular meshes. The first line of the code *Distmesh2D*, the calling syntax, represents inputs and outputs of the Matlab code:

*function* [*p,t*] = *distmesh2d*(*fd,fh,h0,bbox,pfix*)

where the input arguments are as follows:

1. *fd*, the distance function that defines the boundary of the domain.
2. *fh*, the distance function, which controls the convergence of the size of elements. The size of the elements decreases near *fh*.
3. *h0*, the distance between nodes in the initial distribution.
4. *bbox*, the bounding box in which the domain is located.
5. *pfix*, defines nodal points, which are set as fixed points while generating elements.

*Distmesh2D* produces the following outputs:

1. *p*, gives the coordinate of the nodal points.
2. *t*, indicates the connection between the nodes.

Here, coordinates of the nodes on the boundary of a given domain, which are obtained by the BA, are used to define the distance functions *fd* and *fh* for the mesh generator *Distmesh2D*. In addition to the distance functions *fd* and *fh*, *Distmesh2D* has three other inputs: *h0*, *bbox*, and *pfix*. *h0*, the distance between initial nodes, can be set to 1, because the minimum distance between two pixels is 1. *bbox* is equal to the frame size of the imported image (size of the input matrix). The pixels located on the boundaries of the subdomains, extracted by the BA, are defined as fixed points, *pfix*.

*Distmesh2D* can generate both structured and unstructured elements. However, in this study, we set it to create only unstructured elements, because this type of element fits better with our aim for developing models of geometrically complex structures.

### 2.6. Outputs

*WingMesh* generates a *.inp file (i.e., an Abaqus input file) which contains information regarding the coordinates of the nodal points, their connections, type of elements, sections of the domain, and the material properties of sections. Detailed information about *.inp files can be found in the Supplementary Materials (Method S1).

## 3. Graphical User Interface

*WingMesh* was coded in Matlab 2019a, and Matlab App Designer was employed to develop a GUI. This GUI makes the method easy to implement and eliminates the need to know a programming language. The GUI is available in Code S3, and its description is available in Method S1.

## 4. Examples

- Example 1: An in-plane domain

**Figure 3:**
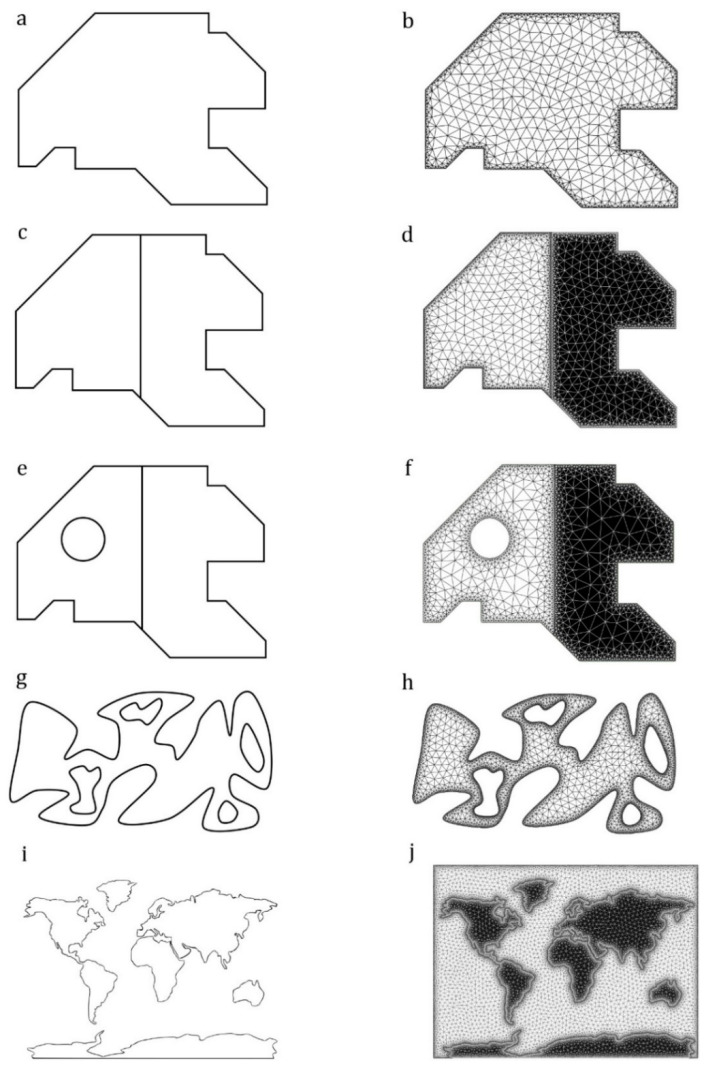
Modeling of in-plane domains. (**a**) Image of a simple in-plane domain. (**b**) The meshed model developed from the image in (**a**). (**c**) Image of an in-plane domain consisting of two subdomains. (**d**) The meshed model developed from the image in (**c**). (**e**) Image of an in-plane domain with two subdomains and a discontinuity. (**f**) The meshed model developed from the image in (**e**). (**g**) Image of an irregular-shaped domain with several discontinuities. (**h**) The meshed model developed from the image in (**g**). (**i**) Image of a complex-shaped in-plane domain with several subdomains. (**j**) The meshed model developed from the image in (**i**).

Figure 3a shows a single in-plane domain with straight-line borders and sharp corners. Figure 3b shows the output model. The *.inp output file developed by the method is available in File S1.

- Example 2: An in-plane domain consisting of two subdomains

Figure 3c illustrates the same domain shown in Figure 3a, which is subdivided into two subdomains. As shown in Figure 3d, *WingMesh* was able to detect the border between the subdomains. The subdomains have meshed separately as two sections of a single model. The generated *.inp file is available in File S2.

- Example 3: An in-plane domain with subdomains and a discontinuity

We added a circular hole within one of the two subdomains of the domain given in the previous example (Figure 3e). After meshing all subdomains, including the discontinuity in the main domain, the elements generated in the discontinuity were removed before the final model was developed (Figure 3f). The *.inp file is available in File S3.

- Example 4: An irregular-shaped in-plane domain with several discontinuities

Figure 3g illustrates an irregular-shaped domain with curved borders, which contains four discontinuities. Previously, it was impossible to model such an irregular domain with complex-shaped discontinuities using the mesh generator *Distmesh2D*. However, the use of the DIP technique enables *WingMesh* to mesh such geometries. Figure 3h shows the meshed model developed based on the given domain. The *.inp file is available in File S4.

- Example 5: A complex-shaped in-plane domain with several subdomains

Figure 3i shows the world map with the irregular shaped continents. The meshed model, which is apparently in good agreement with the given image, is presented in Figure 3j. The *.inp file is available in File S5.

- Example 6: An asymmetric out-of-plane domain with one height maximum and one height minimum

**Figure 4:**
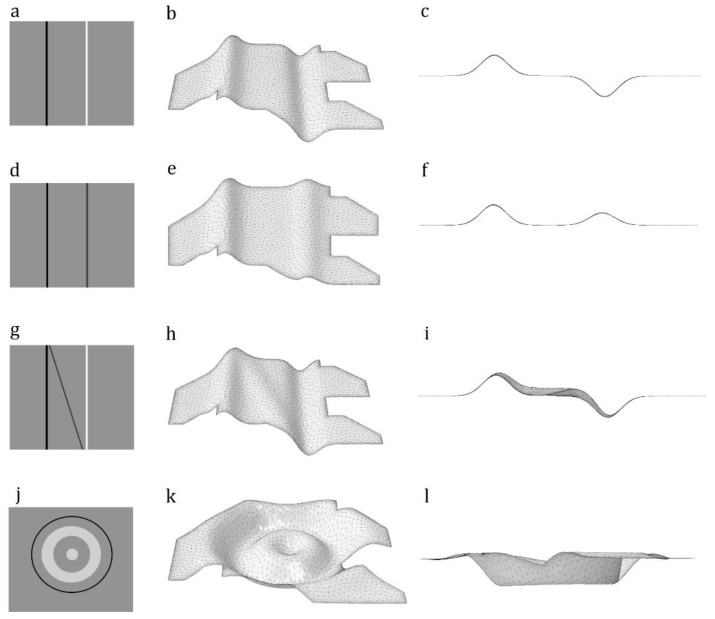
Modeling of out-of-plane domains. The use of different secondary images in combination with the same input image, as shown in Figure 3a, results in the development of models with different corrugated patterns. (**a**,**d**,**g**,**j**) Secondary images contain information on corrugation spots. (**b**,**e**,**h**,**k**) Perspective views of the meshed models created based on the image shown in Figure 3a and secondary images shown in Figure 4a,d,g,j. (**c**,**f**,**i**,**l**) Side views of meshed models.

In this example and the next three cases, we used the domain shown in Figure 3a to develop out-of-plane models with different corrugation patterns. Here, we used the image in Figure 4a as the secondary input image to provide information on the corrugation spots. The grey color in this image indicates regions with zero height. The black and white lines indicate a height maximum and a height minimum, respectively. Figure 4b,c shows the perspective and side views of the meshed model. The *.inp file is available in File S6.

- Example 7: An out-of-plane domain with two height maxima

Figure 4d shows an image with the black and dark grey lines, which represent two height extrema. Using this as a secondary image results in the development of the meshed model shown in Figure 4e,f. The *.inp file is available in File S7.

- Example 8: An out-of-plane domain with two height maxima and a height minimum

In Figure 4g, we added a tilted grey line to those in the secondary image shown in Figure 4a. The grey line is expected to change the corrugation pattern of the meshed model in Figure 4b by adding a region with a height maximum. Figure 4h,i shows the perspective and side views of the model developed using the secondary image in Figure 4g. The *.inp file is available in File S8.

- Example 9: An out-of-plane domain with circumferentially oriented height extrema

In this example, we aimed to test the precision of our method by developing a more complex corrugated domain. In this domain, the corrugation spot is circumferentially oriented, compared with the other domains that had longitudinal corrugations. Figure 4k,l shows the model developed by using Figure 4j. The *.inp file is available in File S9.

- Example 10: A beetle wing

**Figure 5:**
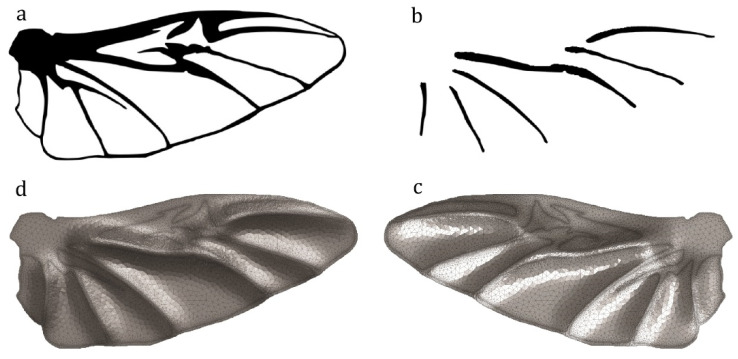
Modeling of the hind wing of the beetle *Allomyrina dichotoma* (Coleoptera: Scarabaeidae). (**a**) Black and white image of the wing. (**b**) The secondary image for generating corrugations showing the location of the elevated veins. (**c**) Dorsal view of the generated model. (**d**) Ventral view of the generated model.

Figure 5a shows the hind wing of a beetle, *Allomyrina dichotoma* (Coleoptera: Scarabaeidae). Figure 5b shows the secondary image that was used for generating the corrugation on the model. The black lines show the location of elevated longitudinal veins in comparison with the membranes. The use of Figure 5b as a secondary input image results in the development of a model that is shown in Figure 5c,d from both dorsal and ventral sides.

## 5. Advantages of *WingMesh*

*WingMesh* offers several advantages over existing manual modeling techniques using commercial software packages, such as CATIA, SolidWorks, Abaqus etc.:

- The application is user-friendly and can remarkably reduce the modeling costs.
- Two-dimensional modeling using *WingMesh* is possible by the use of only an image of a given domain.
- Modeling three-dimensional (3D) out-of-plane domains is simple and can be done by the use of one additional image that contains information on corrugated spots.
- *WingMesh* can develop meshed models of domains that consist of several subdomains and discontinuities.
- *WingMesh* is particularly useful for modeling of a large number of insect wings for comparative investigations.
- Considering the use of computer vision to extract geometric wing features, *WingMesh* is applicable for insect wings that contain a high degree of geometric complexity.
- The input image for *WingMesh* should have only sufficient resolution. This is in contrast to existing tools for extracting morphological features of insect wings using an image, which usually requires high-resolution images at a large size [23,24].

*WingMesh* has improved the applicability of *Distmesh2D*, as listed below:

- Extracting the distance function for complex geometries is a time-consuming and error-prone task, which has been overcome by the use of the computer vision in WingMesh.
- WingMesh generates a *.inp file as the output, which is a frequently used file format.
- WingMesh has an improved ability to mesh structures that contain many discontinuities. This ability was poor in Distmesh2D, especially when dealing with domains with more than one discontinuity.
- In contrast to Distmesh2D, that can mesh domains that have no subdomains, WingMesh is capable of modeling domains with numerous subdomains.
- Compared with Distmesh2D, WingMesh can model out-of-plane domains.

## 6. Applications

The application presented in this study can be used for modeling a wide range of objects in both science and engineering, where a planar FE model is required. For example, models developed by our application could be used to understand the mechanical behavior of biological structures, such as insect wings, plant leaves, etc. (see [25] for more examples). In engineering, it can be employed for FE modeling of plate and shell structures used in aircraft, space crafts, ships, pressure vessels, etc. (see [26,27] for more examples). Our method could also be used in geology and geo-mechanics for the prediction of the mechanical response of complex inhomogeneous rock and concrete structures.

Although *WingMesh* is a promising first step towards the automatic modeling of insect wings, there still remain some other structural features that can be included in a wing model. A few examples of such features are nodus and vein joints, which play key roles in wing deformations both during flight [9,28,29] and at rest (i.e., wing folding [30,31,32]). Hence, as developers of *WingMesh*, we are currently working to develop the next generation of our program, which is able to create wing models with more structural details.

More information on *WingMesh* is available on our website: https://wingquest.org/wingmesh/.

## Supplementary Materials

The following are available online at https://doi.org/10.6084/m9.figshare.12355163: Code S1: The source code of *WingMesh*. Code S2: The source code of the BA. Code S3: The GUI of *WingMesh*. Method S1: The description of the *WingMesh* code. Video S1: Finding the points inside a domain using the BA. Video S2: A tutorial about the use of *WingMesh*. File S1: The **.inp* file of Example 1. File S2: The **.inp* file of Example 2. File S3: The **.inp* file of example 3. File S4: The **.inp* file of example 4. File S5: The **.inp* file of example 5. File S6: The **.inp* file of example 6. File S7: The **.inp* file of example 7. File S8: The **.inp* file of example 8. File S9: The **.inp* file of example 9. File S9: The **.inp* file of example 9. File S10: The **.inp* file of example 10.
